# Supplementary material for: COVID-19-Related Cholangiopathy: Histological Findings
Source: Diagnostics (Basel). 2024 Aug 19;14(16):1804. doi: 10.3390/diagnostics14161804 (PMC11354040; doi:10.3390/diagnostics14161804)
Supplement: Supplementary file 1 [file diagnostics-14-01804-s001.zip › diagnostics-3112106-supplementary.pdf]

Supplementary Materials

COVID-19-Related Cholangiopathy: Histological Findings

Valéria F. A. Borges [1], Helma P. Cotrim [2,*], Antônio Ricardo C. F. Andrade [2], Liliana S. C. Mendes [3], Francisco G. C. Penna [4], Marcelo C. Silva [5], Frederico C. Salomão [6] and Luiz A. R. Freitas [2,7]

[1] Postgraduate Program in Medicine and Health, Federal University of Bahia, Salvador 40026-010, Brazil; valeriaborges@ufba.br
[2] School of Medicine of Bahia, Federal University of Bahia, Salvador 40026-010, Brazil; arcfa01@gmail.com (A.R.C.F.A.); freitas.luizar@gmail.com
[3] Hospital de Base do Distrito Federal, Brasilia 70330-150, Brazil; mendesliliana2@gmail.com (L.S.C.M.)
[4] Department of Internal Medicine, School of Medicine, Federal University of Minas Gerais, Belo Horizonte 31270-901, Brazil; cancelapenna@gmail.com
[5] Hospital e Clínica São Roque, Ipiau 45570-000, Brazil; drmarcelocsilva62@gmail.com
[6] Centro Diagnóstico de Patologia, Uberlandia 38400-110, Brazil; fredericosalomao@yahoo.com.br
[7] Gonçalo Moniz Institute, Oswaldo Cruz Foundation (FioCruz), Salvador 402596-710, Brazil;

* Correspondence: helmacotrim@gmail.com; Tel.: +55-71-99969-6154

Following a severe infection caused by the SARS-CoV-2 virus, confirmed through molecular diagnosis, forty-two patients (27 males, 64%) experienced persistent cholestasis. The demographic and clinical characteristics of all forty-two patients are included in Table S1.

Table S1. Demographic and clinical characteristics of the cohort comprising 42 patients.

N	42
Gender, male, n (%)	27 (64.3%)
Age (years)	
Median [IQR]	60 [53.5-63.7]
Mean ± SD	57.6 ± 10.9
Another etiology for cholestasis	0/42 (0.0%)
Pruritus or jaundice before ICU admission	0/42 (0.0%)
Cholestasis occurs only during occasional sepsis	0/42 (0.0%)
Pregnancy	1/42 (2.38%)
Medications for pre-COVID-19 comorbidities	losartan; metformin; bromazepam; metoprolol; alprazolam; simvastatin; alogliptin; pioglitazone; quetiapine; montelukast; fluticasone; sertraline; omeprazole; levothyroxine; anastrozole; atenolol; vitamin D; zinc; magnesium; amlodipine; acetylsalicylic acid
Hospital stays, days	
Median [IQR]	57 [42-93]
Mean ± SD	69.1± 36.3
Total time of ketamine infusion, days	
Median [IQR]	13 [7.7-22.2]
Mean ± SD	16.7 ± 12.5
D-dimer, ng/mL	
Median [IQR]	591.3 [447.1-756.2]
Mean ± SD	627.5 ± 333.5
CRP, mg/dL	
Median [IQR]	119.8 [44.6 - 191.7]
Mean ± SD	137 ± 118.9
Anticoagulant	15/17 (88.2%)
Antimicrobial	amikacin; anidulafungin; azithromycin; aztreonam; ceftazidime and avibactam; ceftriaxone; fluconazole; linezolid; meropenem; piperacillin and tazobactam; polymyxin; teicoplanin; trimethoprim-sulfamethoxazole; tigecycline; ertapenem; levofloxacin; ciprofloxacin; moxifloxacin; clavulanate; micafungin; vancomycin; gentamicin; acyclovir; ampicillin; oxacillin; imipenem
Antiviral	0/42 (0.0%)
Follow-up, days	
Median [IQR]	796 [429.2-931.5]
Mean ± SD	705.3 ± 345.5
Evolution to death	12/42 (28.57)
Liver transplant	1/42 (2.4%)

IQR: interquartile range; SD: standard deviation; U/L: units per liter; mg/dL: milligrams per deciliter.

Table S2 provides a detailed overview of the histopathological features observed in ten patients with COVID-19-related cholangiopathy livers. The findings are categorized across various components, such as portal tracts, biliary tree, and hepatic parenchyma, focusing on cholestasis. Each parameter is graded based on severity, where "1" indicates mild, "2" indicates moderate, and "3" indicates severe, with "ND" denoting not done. The specific histopathological findings include Portal tracts: The assessment consists of the number of tracts, degree of inflammation, and presence of lymphocytes, neutrophils, eosinophils, fibrosis, and septal fibrosis.

Biliary tree: Evaluation includes the degree of dystrophy, vacuolization, ductular changes, and presence of bile plugs. Hepatic parenchyma: Observations on canalicular and hepatocyte vacuolization, CK7+ ductular metaplasia, and instances of necrosis/apoptosis are included.

Table S2. Histopathological liver findings in COVID-19-related cholangiopathy in each of 10 cases.

	**Portal tracts**							**Biliary tree**				**Hepatic parenchyma**			
												**Cholestasis**			
**Case**	**No.**	**Inflam.**	**Lyt**	**Neu**	**Eos**	**Fib.**	**Septal Fib.**	**Dyst.**	**Vac.**	**Duct.**	**Bile plugs**	Can.	Hep.	CK7 + Duct. Metap.	Nec./Apop.
1	15	2	2	1	Rare	2	1	3	3	2	0	3	3	ND	1
2	25	2	2	2	Rare	2	2	2	2	2	1	1	1	3	1
3	30	2	2	1	Rare	2	2	2	1	2	0	1	1	1	1
4	22	1	2	1	Rare	3	2	2	2	3	1	3	3	2	1
5	23	2	2	1	Rare	2	2	3	3	2	2	2	2	2	1
6	29	1	2	1	0	1	0	2	0	1	2	1	1	1	1
7	26	2	2	1	Rare	3	3	3	1	3	2	1	1	2	1
8	18	2	2	1	0	2	0	2	1	2	2	2	3	2	Foci of biliary infarcts
9	32	1	2	1	0	2	2	1	1	3	0	1	1	ND	1
10	29	2	1	1	0	1	1	3	2	3	0	0	0	ND	1

No.: Number of portal tracts; Inflam.: Inflammation; Lyt: Lymphocytes; Neu: Neutrophils; Eos: Eosinophils; Fib.: Fibrosis; Septal Fib.: Septal fibrosis; Dyst.: Dystrophy; Vac.: Vacuolization; Duct.: Ductular; Can.: Canalicular; Hep. Vac.: Hepatocyte Vacuolization; CK7+ Duct. Metap.: CK7+ Ductular Metaplasia; Nec./Apop.: Necrosis/Apoptosis; "1" for mild; "2" for moderate; "3" for severe; and "ND" for not done; CK: Cytokeratin.

The distribution of different types of inflammatory cells is demonstrated in Figure S1. This figure illustrates the varying levels of inflammatory cell types found in the liver tissue of ten patients with COVID-19-related Cholangiopathy, including eosinophils, neutrophils, lymphocytes, and overall inflammation. These levels are categorized and represented by distinct colors: Blue (None), Orange (Rare), Gray (Mild), Yellow (Moderate), and Light Blue (Severe).

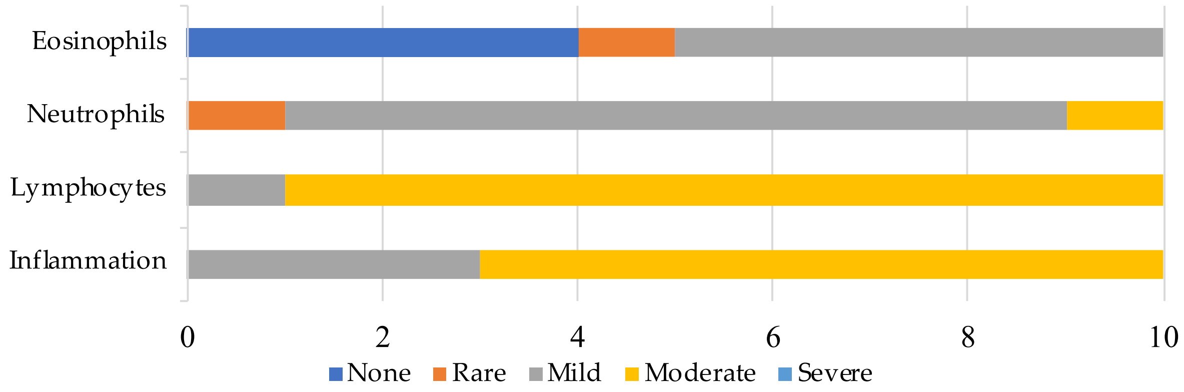

Figure S1. Severity distribution of inflammatory cells.

Among the 12 patients who died during the follow-up period, 11 (91.7%) died due to liver-related causes, specifically decompensation of advanced chronic liver disease, while 1 (8.3%) died due to non-liver-related causes, as detailed in *Table S3*.

Table S3. Characteristics of the 12 deceased patients within the cohort of 42.

N	12
Gender, male, n (%)	7 (58.3%)
Age, years	
Median [IQR]	65.5 [63.7-70.5]
Mean ± SD	66.2 ± 7.5
Hospital stays, days	
Median [IQR]	45 [38-82]
Mean ± SD	63.5 ± 38.9
Time from SARS-CoV-2 infection to death, days	
Median [IQR]	213.5 [133.2-529.2]
Mean ± SD	362.9 ± 328.4

IQR: interquartile range; SD: standard deviation.

Table S4 provides the demographic characteristics of the eight surviving patients among the ten who underwent liver biopsy.

Table S4. Demographic characteristics of the eight surviving patients among the ten who underwent liver biopsy.

N	8
Gender, male, n (%)	4 (50%)
Age, years	
Median [IQR]	56 [50-60]
Mean ± SD	53.6 ± 13.1
Hospital stays, days	
Median [IQR]	56 [37-71.7]
Mean ± SD	65.7 ± 37.5
Follow-up, days	
Median [IQR]	834 [784.7-903.5]
Mean ± SD	775.6 ± 221.7
Liver transplant, n (%)	0 (0.0%)
Evolution to biliary cirrhosis, n (%)	1 (12.5%)
Minimal changes in liver tests, n (%)	2 (25.0%)
Severe cholestasis, n (%)	5 (63.5%)

Minimal changes in liver tests: maintenance of elevated liver enzymes less than two times the upper limit of normal; Severe cholestasis: Alkaline phosphatase or gamma-glutamyl transferase greater than five times the upper limit of normal; IQR: interquartile range; SD: standard deviation.

Our study observed that half of the samples exhibited swelling characterized by the endothelial cells' swollen, vacuolated appearance. This feature was incidentally found in 50% of cases, specifically in 5 samples. Our purpose was to report the occurrence of this swelling rather than to quantify it. This feature was not previously described in cases of secondary sclerosing cholangitis in critically ill patients (SSC-CIP) before the COVID-19 pandemic. The mechanism behind this alteration in SARS-CoV-2 infection has yet to be established. It may be due to a direct effect of the virus on the endothelial cells, or it could be a physical manifestation of endothelial dysfunction associated with microthrombosis. Interestingly, we found no correlation between the levels of hepatic enzymes or total bilirubin and the swelling of endothelial cells in branches of the hepatic artery (Table S5).

Table S5. Correlation of liver enzyme levels and total bilirubin with endothelial cell swelling in branches of the hepatic artery

	Spearman's rho	p-value	FHP CI95%		BW CI95%		CC CI95%	
			Lower	Upper	Lower	Upper	Lower	Upper
AST vs. swelling	-0,211	0,559	-0,752	0,500	-0,745	0,489	-0,723	0,452
ALT vs. swelling	-0,368	0,295	-0,818	0,359	-0,818	0,362	-0,798	0,310
ALP vs. swelling	-0,026	0,942	-0,658	0,627	-0,645	0,614	-0,617	0,583
GGT vs. swelling	0,368	0,295	-0,359	0,818	-0,362	0,818	-0,310	0,798
Bilirubin vs. swelling	0,109	0,765	-0,574	0,702	-0,561	0,692	-0,528	0,668

FHP CI95%: Fisher-Hayter Procedure Confidence Interval at 95%; BW CI95%: Bonferroni-Welch Confidence Interval at 95%; CC CI95%: Carlin-Ciuffo Confidence Interval at 95%; AST: Aspartate Aminotransferase; ALT: Alanine Aminotransferase; ALP Alkaline Phosphatase; GGT: Gamma-Glutamyl Transferase; vs.: versus; The liver enzymes and total bilirubin were expressed as multiples of the upper limit of normal.

There was no significant correlation between the histological findings and the blood coagulation parameters we had available. This information is included in Table S6.

Table S6. Blood coagulation parameters of the cohort of 42 patients.

D-dimer, ng/mL	
Median [IQR]	591.3 [468-750.4]
Mean ± SD	644.4 + 355.2
Anticoagulant use, n (%)	15/17 (88.2%)

IQR: interquartile range; SD: standard deviation.

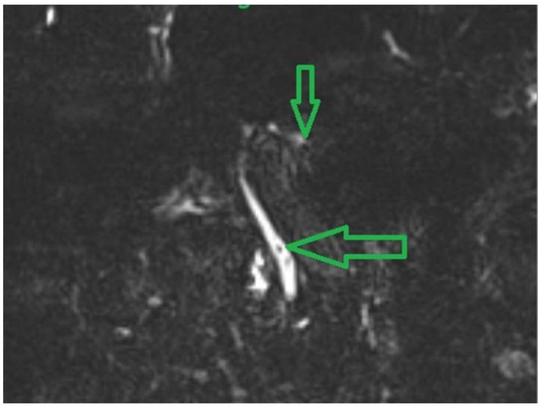

Figure S2. This magnetic resonance cholangiopancreatography image was captured 202 days after the COVID-19 infection. It highlights a filling defect in the distal common bile duct, indicating the presence of a biliary cast.

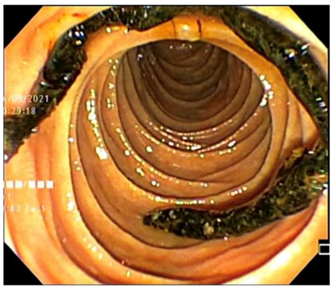

Figure S3. An endoscopic photograph of the duodenal second portion exhibiting extracted casts via Endoscopic Retrograde Cholangiopancreatography 233 days following the onset of COVID-19.

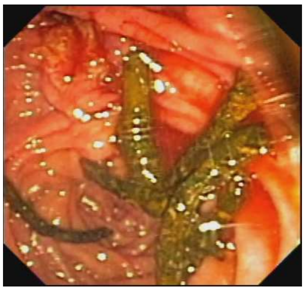

Figure S4. An endoscopic image of the duodenal second portion illustrates biliary casts that were extracted via endoscopic retrograde cholangiopancreatography. This particular case was observed 327 days following the COVID-19 diagnosis.
